# GLI3 is Inhibited by miR-143-3p and Attenuates Septic-induced Lung Injury and Inflammation by Targeting SFRP1

**DOI:** 10.2174/0113862073354161250220121414

**Published:** 2025-03-10

**Authors:** Minqing Ma, Haixia Han, Xiaoyan Luo, Jiakai Lin, Bin Sun

**Affiliations:** 1 Department of Emergency, Binzhou Medical University Hospital, Binzhou 256600, China

**Keywords:** Septic lung injury, transcription factor, miRNA, LPS, miR-143-3p, inflammation

## Abstract

**Objectives:**

Transcription factors (TF) are the central regulatory hubs of signaling pathways in eukaryotic cells. Here, we explored the abnormal expression of TF in septic-induced lung injury by sequencing.

**Methods:**

The levels of target proteins were detected using Western Blot and Elisa. Cell function was evaluated using CCK8 and transwell assays. A double luciferase reporter assay was performed to detect interactions between target molecules.

**Results:**

We found that TF *glioma-associated oncogene (GLI) family zinc finger 3* (*GLI3*) was abnormally low expressed in a lipopolysaccharide (LPS) induced acute lung injury (ALI) cell model. In an *in vitro* model, *GLI3* overexpression promoted the proliferation and migration and inhibited apoptosis of lung epithelial cells in LPS-induced inflammatory environment. Importantly, *GLI3* overexpression inhibited the secretion of inflammatory factors IL-1β, IL-6, and TNF-α. Additionally, miR-143-3p inhibited the expression of *GLI3*. MiR-143-3p inhibitor alleviated the cell damage caused by LPS, while knocking down *GLI3 counteracted* this effect, indicating that miR-143-3p downregulated *GLI3* and inhibited its anti-inflammatory effect. *Secreted frizzled related protein-1* (*SFRP1*) was upregulated in LPS-treated cells and *SFRP1* promoter interacted with *GLI3*, suggesting that *SFRP1* was a target of TF *GLI3*. Co-transfection with *GLI3* knockdown and *SFRP1* overexpression plasmids attenuated the secretion of inflammatory factors IL-1β, IL-6, and TNF-α caused by *GLI3* knockdown in LPS-treated cells, indicating that *SFRP1* plays an anti-inflammatory role as a *GLI3* target in the ALI cell model.

**Conclusion:**

miR-143-3p caused degradation of *GLI3* mRNA and thus inhibited the transcription of *SFRP1*, leading to decreased proliferation and increased levels of inflammatory factors, providing new potential targets for the clinical diagnosis and treatment of ALI.

## 1 INTRODUCTION

Sepsis is a common complication severe infection, trauma, burns, and major surgery [1]. It is a life-threatening organ dysfunction caused by an uncontrolled host response to infection, with the characteristics of high morbidity, high mortality, and poor long-term prognosis. It remains one of the main causes of death in intensive care unit patients [2]. Sepsis causes acute damage to multiple organs, particularly the lungs. Acute lung injury (ALI) is one of the most common complications of sepsis and is characterized by progressive and intolerable hypoxemia [3]. Further development of ALI can lead to acute respiratory distress syndrome (ARDS). Current treatment strategies have no significant effect on reducing the mortality of patients with ALI, and the mortality in ARDS patients remains close to 40%. Therefore, there is an urgent need to explore the molecular mechanisms underlying the occurrence and development of ALI to develop more effective treatments.

Transcription factors (TF) are the central regulatory hub of signaling pathways in eukaryotic cells [4]. Abnormally expressed TFs are involved in the pathogenesis and progression of many human diseases, including diabetes, inflammation, cardiovascular diseases, and cancers, and have huge clinical therapeutic potential [5-7]. Small-molecule drugs targeting specific TFs can affect TF function by modulating the interaction between TFs and DNA or by interfering with TF dimerization [8, 9]. TFs are regulatory hubs of signaling pathways, so targeting these proteins may be more specific than traditional targets. However, due to the complexity and dynamics of TF networks, elucidating the upstream and downstream regulatory networks of TFs is essential for future clinical applications.

In the present research, lung epithelial cells were treated with lipopolysaccharide (LPS) to simulate sepsis-induced ALI, and an abnormal expression profile of TF was obtained by sequencing. Based on these results, we hypothesized that *GLI3* is involved in the LPS-induced lung cell injury as a TF. *GLI3* is a key TF in the Hedgehog (Hh) signaling pathway and is involved in the regulation of angiogenesis, tumor progression, and apoptosis [10-12]. In addition, *GLI3* plays a role in mouse lung development [13]. However, the role of *GLI3* in lung injury has not been reported. This study aimed to explore the role and specific mechanisms of *GLI3* on cell proliferation and apoptosis in ALI cells. Our research enriches our understanding of the development mechanism of ALI and explores new research focuses and potential therapeutic targets for sepsis-induced ALI.

## 2 MATERIALS AND METHODS

### 2.1 Cell line and Treatment

The human lung epithelial cell line BEAS-2B was purchased from Mingzhoubio (Ningbo, China) and cultured in DMEM with 10% FBS at 37 °C with 5% CO_2_. Cells were treated with 0.5 μg/ml LPS (L8880, Solarbio Science and Technology Ltd., Beijing, China) for 24 h to simulate cell injury [14]. The *GLI3* overexpression plasmid was synthesized by GENECHEM (Shanghai, China). Transfection was performed using Lipofectamine 2000 (11668019, Thermo Fisher Scientific, USA).

### 2.2 Real-time Fluorescence Quantitative PCR (qPCR)

Total RNA was extracted using a Total RNA Extraction Kit (R1200, Solarbio Science and Technology Ltd., Beijing, China) and then reverse transcribed into cDNA using a Universal RT-PCR Kit (RP1100, Solarbio Science and Technology Ltd., Beijing, China; CW2141S, CoWin Biotech Co. Ltd., Beijing, China) according to the manufacturer's instructions. UltraSYBR Mixture (CW0957H and CW2142S, CoWin Biotech Co. Ltd., Beijing, China) was used for qPCR detection. *GAPDH* was used as a loading control. The expression of target mRNA was analyzed by 2^-ΔΔCT^ method [15]. Primers used in this research are listed in Table **1**. QPCR was performed using a Real-Time Quantitative Thermal Cycler (FTC-3000, FUNGLYN biotech, Canada, made in 2015).

### 2.3 Cell Proliferation Assay

BEAS-2B cells were seeded into a 96-well plate after treatment or transfection. Cell proliferation was detected using a Cell Counting Kit (CCK8) reagent (CK04, Solarbio Science and Technology Ltd., Beijing, China) every 24 h according to the manufacturer's instructions [16]. Cells were incubated with 10 μl of CCK8 for 1.5 h at 37 °C. The optical density (OD) was measured at 450 nm using a microplate reader (TECAN, INFINITE 200 Pro, Austria, made in 2020).

### 2.4 Transwell for Migration Assay

Transwell chambers were purchased from Invitrogen (140652, Carlsbad, CA). After treatment or transfection for 24 h, 5000 BEAS-2B cells in serum-free medium were transferred to the upper chamber, and a medium with 10% FBS was added to the lower chamber. After 24 h of incubation, cells were stained with 0.1% crystal violet for 5 min [17] and photographed at 100x magnification. Migrating cells were counted in three random fields.

### 2.5 Western Blot

The expression levels of GLI3, Caspase3-P17, and BCL2 were detected by western blot [7, 18]. After LPS treatment or transfection for 24 h, the total protein was extracted using RIPA buffer. The samples were electrophoresed using SDS-PAGE and transferred to a polyvinylidene fluoride (PVDF) membrane. Immune response was and assessed after blocking with fat-free milk for 40 min at room temperature. The membrane was then incubated with the primary antibody for 1 h at room temperature, followed by incubation with the secondary antibody for 1 h at room temperature. The antibodies against GLI3 (1:1000, ab181130), Caspase3-P17 (1:5000, ab32351), and BCL2 (1:2000, ab32124) used in this research were all purchased from Abcam (Cambridge, UK). An enhanced chemiluminescence (ECL) kit was used for luminescence (08-110, Merck-Millipore, Germany). The results were analyzed using ImageJ software.

### 2.6 Elisa

Protein levels in the supernatant of BEAS-2B cells treated for 24 h were detected according to the manufacturer's instructions. Elisa kits for IL-1β (ab214025), IL-6 (ab178013), and TNF-α (ab181421) were purchased from Abcam (Cambridge, UK) [19]. After the immune reaction, an OD value at 450 nm was detected using a microplate reader (TECAN, INFINITE 200 Pro, Austria, made in 2020).

### 2.7 Double Luciferase Reporter Assay

The binding of the *SFRP1* promoter to *GLI3* was confirmed using a double luciferase reporter assay [20]. The cells were divided into three groups for transfection. The fluorescent plasmid with *SFRP1* promoter region (2000 bp) and *GLI3* overexpression plasmid were co-transfected into BEAS-2B cells (pGL3-SFRP1 promoter-WT group); the fluorescent plasmid containing truncated *SFRP1* promoter region (500bp) and *GLI3* overexpression plasmid were co-transfected into BEAS-2B cells (pGL3-SFRP1 promoter-MUT group). *GLI3* overexpression plasmid and pGL3 were co-transfected into BEAS-2B cells as a control (pGL3 group). The plasmids used in the double luciferase reporter assay were synthesized by GENECHEM (Shanghai, China). After transfection for 24 h, the firely lueiferase (F) and Renilla luciferase (R) values were detected using a microplate reader (TECAN, INFINITE 200 Pro, Austria, made in 2020), and the relative luciferase activity (F/R) was calculated.

### 2.8 Statistical Analyses

Data are presented as mean ± standard deviation (SD). One-way analysis of variance (ANOVA) was performed for multiple comparisons. The data were analyzed and graphed using GraphPad Prism 8.0 (GraphPad, San Diego, CA, USA). All the experimental results involved in the analysis were obtained from three replicates. *P* < 0.05 were considered statistically significant.

## 3 RESULTS

### 3.1 LPS Treatment Affects the RNA Expression Profile of Lung Epithelial Cells

**Fig. (1):**
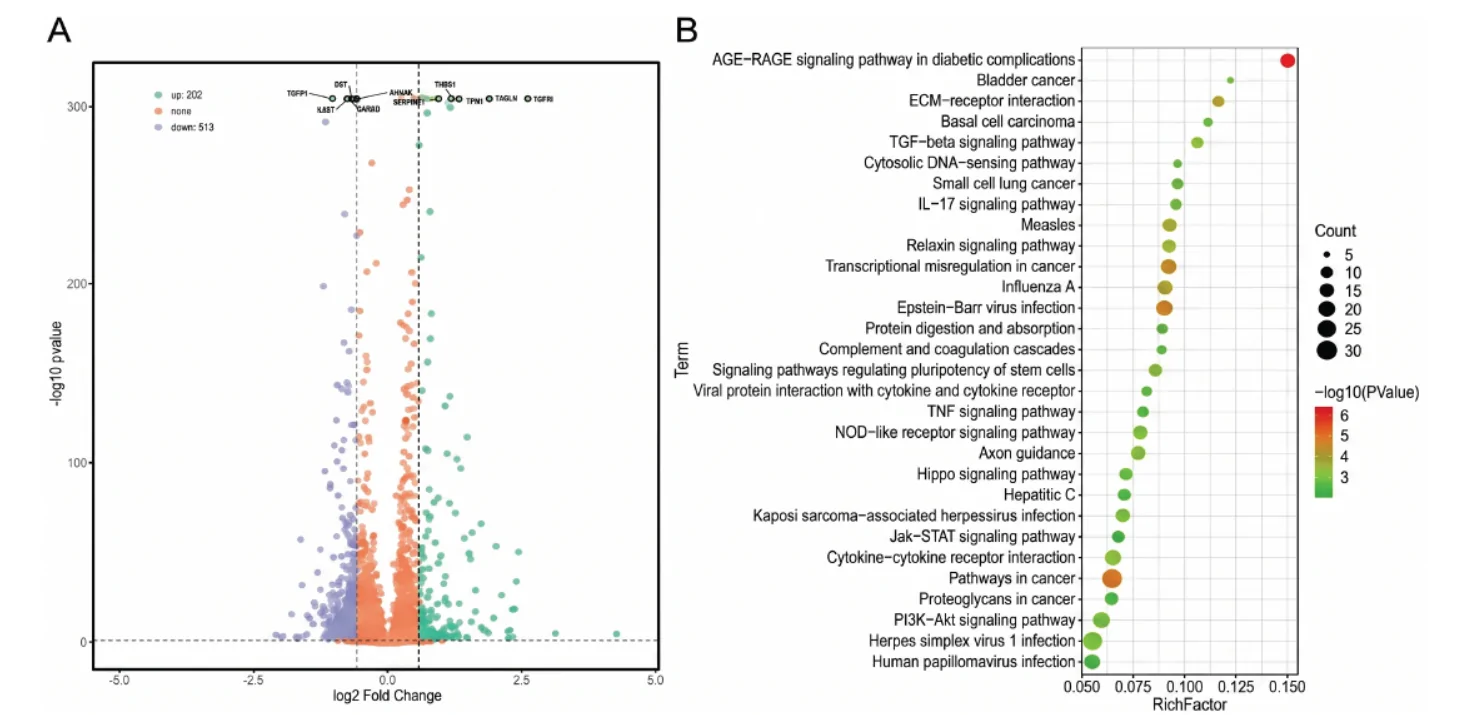
Transcriptome sequencing for ALI cell model. (**A**) Human lung epithelial cell line BEAS-2B was treated with 0.5 μg/ml LPS to construct ALI lung epithelial cell model, and then the cell model was sequenced. Volcanic map was plotted for the differentially expressed genes. (**B**) KEGG enrichment analysis was performed for the differentially expressed genes. The size of the dot indicates the number of differentially expressed genes in the KEGG, while the color of the dot corresponds to p value; Richfactor=n(differential genes enriched in the pathway)/n(annotated genes).

The RNA expression profile of the ALI cell model was analyzed to investigate the molecular mechanism of ALI progression. The human lung epithelial cell line BEAS-2B was treated with 0.5 μg/ml LPS to construct an ALI lung epithelial cell model. The differential gene expression profile was obtained through sequencing. A total of 202 significantly up-regulated genes and 513 significantly down-regulated genes were identified (Fig. **1A**). Pathway enrichment analysis showed that differentially expressed genes were enriched in signaling pathways related to inflammation, proliferation, and apoptosis, including JAK/STAT, PI3K/AKT, TNF, and IL-17 signaling pathways (Fig. **1B**).

### 3.2 TF *GLI3* Inhibits the Cell Injury Induced by LPS in BEAS-2B Cells

**Fig. (2):**
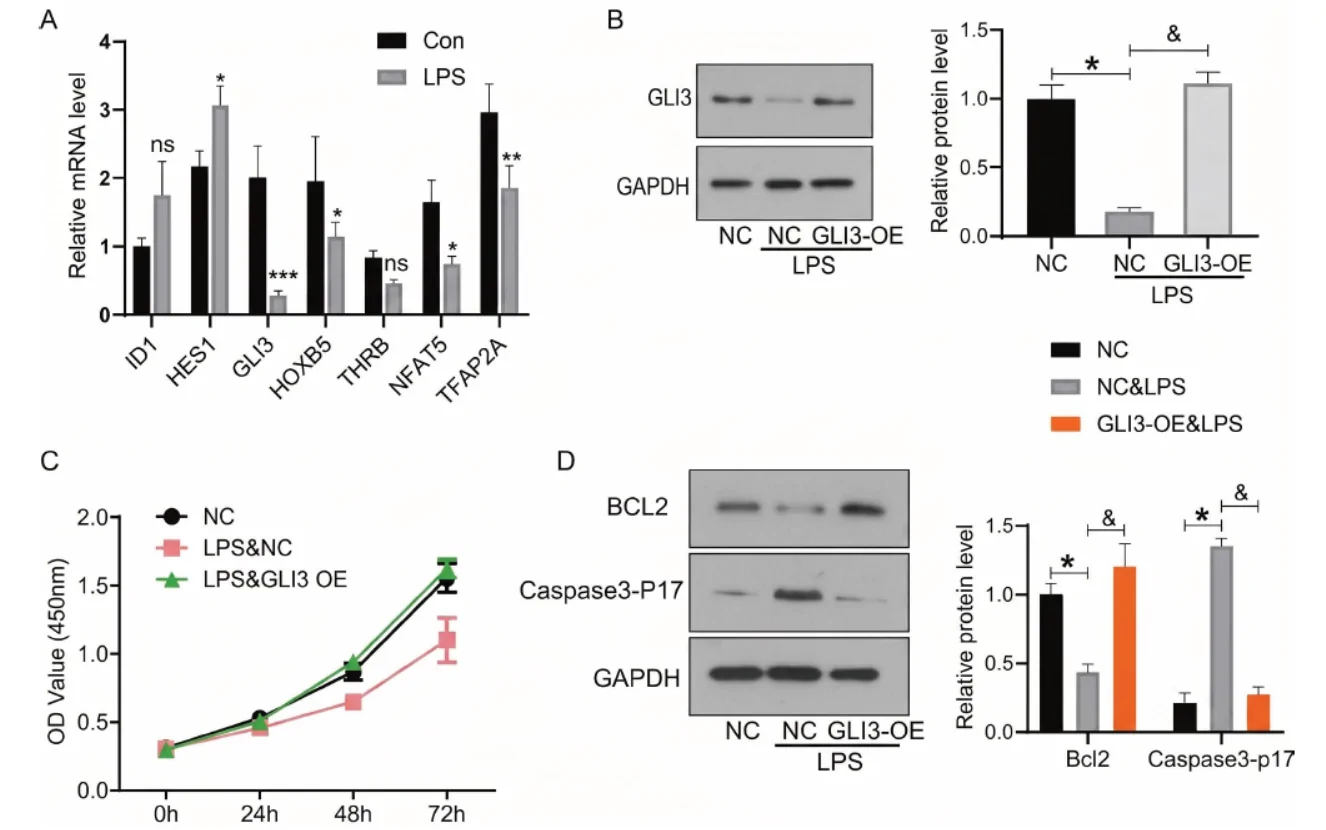
*GLI3* inhibits the proliferation of LPS treated BEAS-2B cells. (**A**) The expression levels of TFs were detected by qPCR in 0.5 μg/ml LPS treated BEAS-2B cells (LPS). The BEAS-2B cells in routine cultivation were used as the control group (Con). (**B**) The overexpression plasmid of *GLI3* was transfected into 0.5 μg/ml LPS treated BEAS-2B cells (GLI3-OE) with the pcDNA3.1 as a negative control (NC). GLI3 expression was detected by western blot. (**C**) Cell proliferation was detected by CCK8 at 24 h intervals. (**D**) The apoptotic proteins were detected by western blot. *p<0.05 compared with NC group; **p<0.01 compared with NC group; ***p<0.001 compared with NC group; ^&^*p<*0.05 compared with 0.5 μg/ml LPS treated NC group.

According to the results of sequencing, a total of 7 differentially expressed TFs with |log2 FC|>1 was intercepted. These 7 TFs levels were detected in BEAS-2B cells. *GLI3* was down-regulated most significantly in 0.5 μg/ml LPS-treated BEAS-2B cells compared with the control group (Fig. **2A**).

**Fig. (3):**
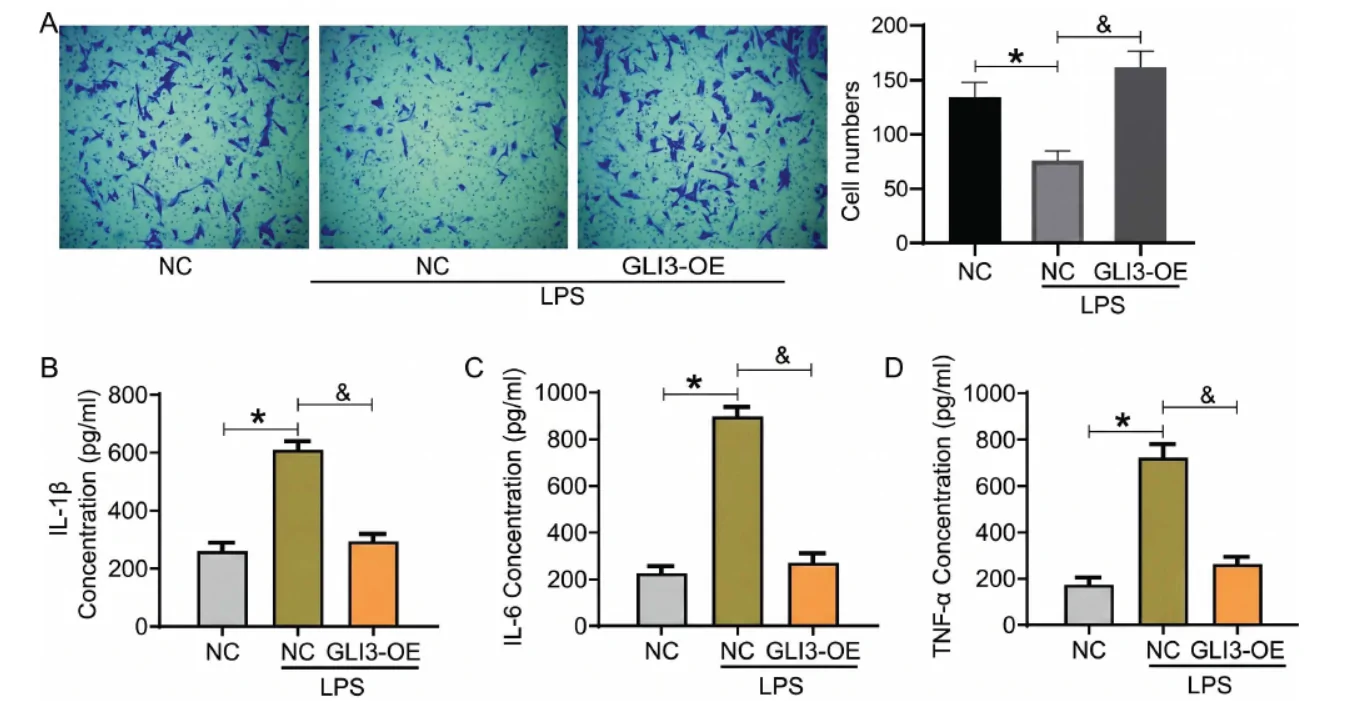
*GLI3* inhibits the migration and secretion of inflammatory factors in LPS treated BEAS-2B cells. (**A**) Cell migration was detected using transwell assay after the transfection for 24 h. (**B-D**) After 24 h of transfection, the cell supernatant was collected, and the levels of IL-6, TNF-α and IL-1β were detected by ELISA assay. *p<0.05 compared with NC group; ^&^*p<*0.05 compared with 0.5 μg/ml LPS treated NC group.

To investigate the specific role of *GLI3*, *GLI3* overexpression plasmid was synthesized. *GLI3* expression increased significantly after transfection of *GLI3* overexpression plasmid (GLI3-OE group) (Fig. **2B**). In CCK8 assay. the OD value of LPS-treated cells decreased significantly, while increasing to the control level after *GLI3* overexpression (Fig. **2C**). Importantly, In LPS group, apoptosis factor Caspase3-p17 was up-regulated, while anti-apoptosis factor BCL2 was down-regulated; after the transfection of *GLI3* overexpression plasmid, the levels of Caspase-p17 and BCL2 were rescued to the control level (Fig. **2D**). Transwell results showed that the number of migrating cells (76±9) decreased significantly compared with the control after LPS treatment (134±14), which was blocked by *GLI3* overexpression (161±15) (Fig. **3A**).

As shown in Fig. (**3B**-**D**), *GLI3* overexpression could effectively prevent the secretion of inflammatory factors (IL-1β, IL-6, and TNF-α) induced by LPS. These results suggested that *GLI3* overexpression protected BEAS-2B cells from LPS-induced cell injury.

### 3.3 MiR-143-3p Inhibited *GLI3* Expression

**Fig. (4):**
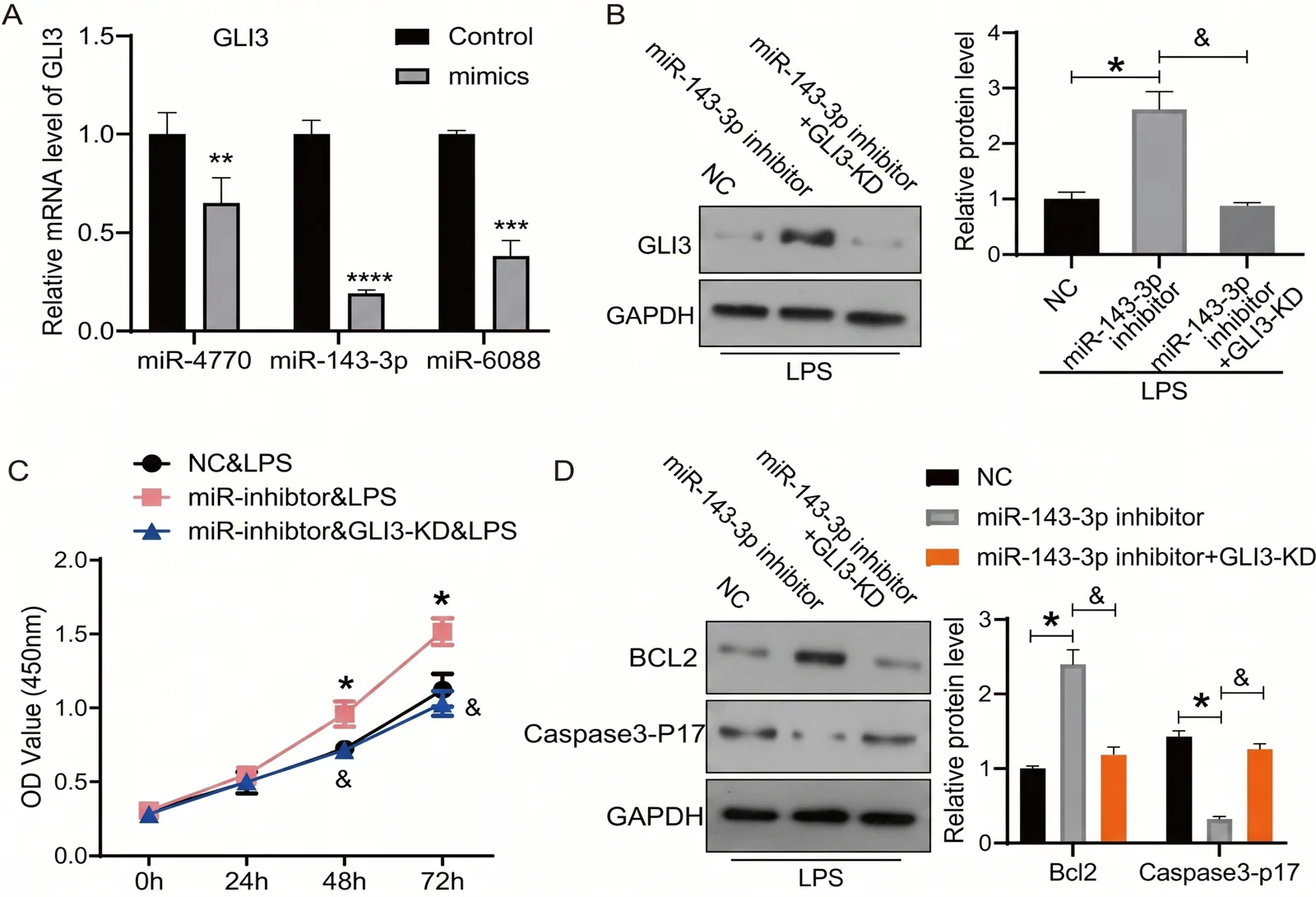
MiR-143-3p inhibitor attenuates the LPS induced apoptosis through targeting *GLI3*. (**A**) GLI3 expression was detected by qPCR after transfection of mimics (miR-4770, miR-143-3p or miR-6088) into BEAS-2B cells for 24 h. (**B**) The expression of GLI3 was detected by western blot in NC, miR-143-3p inhibiter and miR-143-3p inhibiter+GLI3-KD groups. All the cells were treated with 0.5 μg/ml LPS. (**C**) Cell proliferation was detected by CCK8 at 24 h intervals. (**D**) The apoptotic proteins were detected by western blot. *p<0.05 compared with NC group; **p<0.01 compared with NC group; ***p<0.001 compared with NC group; ****p<0.0001 compared with NC group; ^&^*p<*0.05 compared with miR-143-3p inhibiter group.

We analyzed microRNAs (miRNAs) that bound to *GLI3* using Targetscan, a dataset focused on the prediction of miRNA-mRNA binding [21]. MiR-4770, miR-143-3p, and miR-6088 contained binding sites with *GLI3*. As shown in Fig. (**4A**), the mimics of miR-4770, miR-143-3p, and miR-6088 inhibited the expression of *GLI3*. MiR-143-3p had the most significant inhibitory effect on *GLI3*. After transfection of miR-143-3p inhibitor, *GLI3* expression increased significantly in LPS-treated cells (Fig. **4B**). Co-transfection of miR-143-3p inhibitor and *GLI3* siRNA resulted in a significant decrease in *GLI3* expression compared with miR-143-3p inhibitor alone (Fig. **4B**).

### 3.4 MiR-143-3p Inhibitor Attenuates LPS-induced Inflammation and Injury by Targeting *GLI3*

**Fig. (5):**
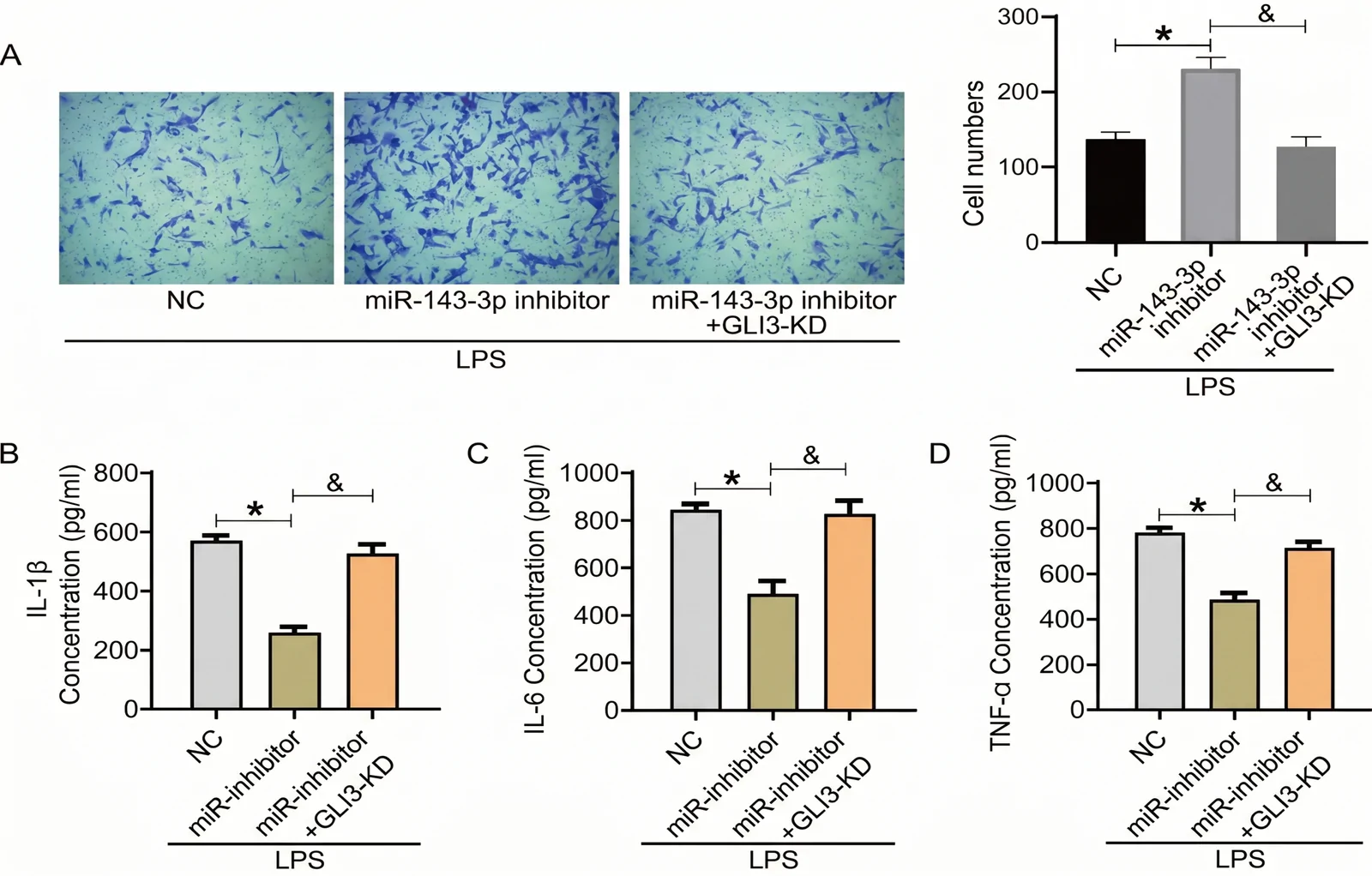
MiR-143-3p inhibitor reduces inflammatory factors secretion induced by LPS through targeting *GLI3*. (**A**) Cell migration was detected using transwell assay after the transfection for 24 h. The cell supernatant was collected After 24 h of transfection, and the levels of IL-6 (**B**), TNF-α (**C**) and IL-1β (**D**) were detected by ELISA assay. *p<0.05 compared with NC group; ^&^*p<*0.05 compared with miR-143-3p inhibiter group.

As shown in Fig. (**4C**), miR-143-3p inhibitor promoted the proliferation, while *GLI3* knockdown reduced the proliferation compared with miR-143-3p inhibitor alone. MiR-143-3p inhibitor significantly down-regulated the level of Caspase-p17, but up-regulated the level of BCL2. These effects were reversed by *GLI3* knockdown (Fig. **4D**). In addition, cell migration showed a similar trend. Cell migration was promoted by miR-143-3p inhibitor, and reduced by the co-transfection of *GLI3*-siRNA (NC: 138±9; miR-143-3p inhibitor: 231±15; miR-143-3p inhibitor+GLI3-KD: 127±13) (Fig. **5A**).

Importantly, the expression levels of inflammatory factors IL-1β (Fig. **5B**), IL-6 (Fig. **5C**), and TNF-α (Fig. **5D**) were significantly decreased in miR-143-3p inhibitor-treated cells. *GLI3* knockdown reversed the inhibition of inflammatory factors induced by miR-143-3p inhibitor. These results proved that miR-143-3p inhibitor alleviated the cell damage caused by LPS, and *GLI3* knockdown counteracted this effect. These data indicated the role of *GLI3* as a regulatory target of miR-143-3p.

### 3.5 *SFRP1* as a Target of *GLI3* Plays an Anti-inflammatory Role in the ALI Cell Model

**Fig. (6):**
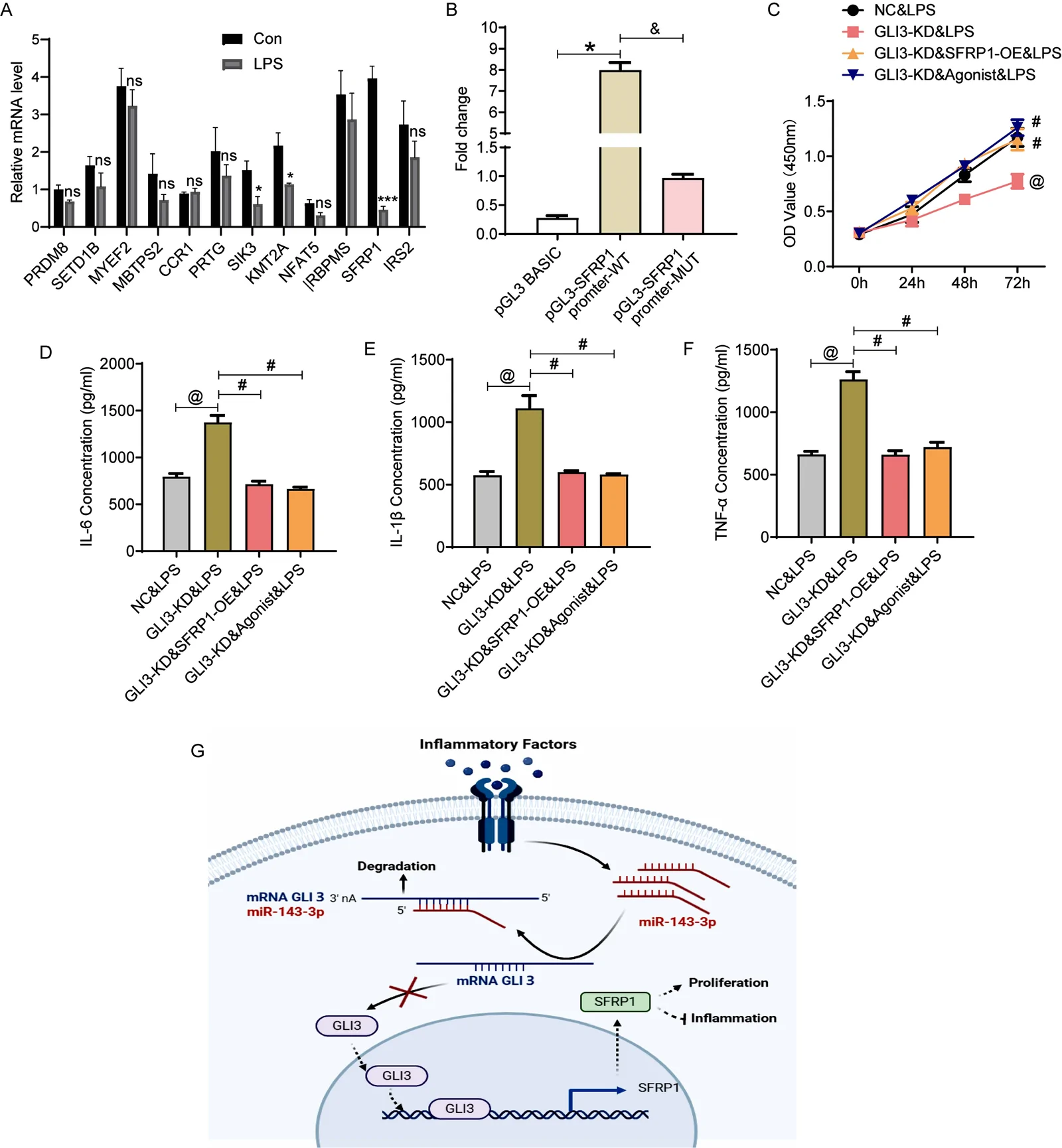
*SFRP1* operates as *GLI3* target and exerts an anti-inflammatory effect in ALI cell model. (**A**) QPCR was used to detect the expression of predicted targets of *GLI3*. * *p<*0.05; ***p<0.001 (**B**) Double luciferase assay was performed to confirm the binding of *GLI3* with *SFRP1* promoter. (**C**) Cell proliferation was detected using CCK8 assay. (**D-F**) ELISA was used to detect the level of inflammatory factors. (**G**) Diagram of the regulation mode of miR-143-3p/*GLI3*/*SFRP1* in inflammatory response. *p<0.05 compared with pGL3 BASIC group; ^&^*p<*0.05 compared with pGL3-SFRP1 promoter-WT group; ^@^*p<*0.05 compared with NC&LPS group; ^#^*p<*0.05 compared with GLI3-KD&LPS group.

The sequencing results showed that 12 of the predicted targets of *GLI3* were significantly down-regulated. As shown in Fig. (**6A**), 10 candidate genes were down-regulated, of which *SFRP1* down-regulated most significantly. Therefore, we focused on analyzing the transcriptional regulation of *SFRP1* by *GLI3* using dual luciferase reporter gene analysis. *SFRP1* promoter plasmid (pGL3-SFRP1 promoter-WT) significantly increased the fluorescence intensity compared with the control cells (pGL3); while the fluorescence intensity was significantly decreased by the transfection of truncated *SFRP1* promoter (pGL3-SFRP1 promoter-MUT) (Fig. **6B**).

Then, we investigated the specific role of *SFRP1* in LPS-treated BEAS-2B cells. Overexpression of *SFRP1* attenuated GLI3 knockdown-induced inhibition on cell proliferation (Fig. **6C**). Co-transfection of *GLI3* knockdown and *SFRP1* overexpression plasmids attenuated the secretion of inflammatory factors (IL-1β, IL-6 and TNF-α) induced by *GLI3* knockdown in LPS-treated cells (Fig. **6D**-**F**).

### 3.6 Tofacitinib (JAK Inhibitor) Reverses *GLI3* Knockdown-induced Cell Injury in ALI Cell Model

According to KEGG results, differential genes were significantly enriched in JAK / STAT signaling pathway. Tofacitinib is a clinical JAK inhibitor (agonist) currently used in the treatment of rheumatoid arthritis. Tofacitinib reduced the inhibition of proliferation induced by *GLI3* knockdown in ALI cell model (Fig. **6C**). The increase in cytokine secretion caused by *GLI3* knockdown is significantly inhibited by tofacitinib (Fig. **6D**-**F**).

Our data indicated that miR-143-3p combines with *GLI3* mRNA to induce its degradation, resulting in the decrease of *GLI3* protein level. Then, SFRP1 was downregulated and decreased the proliferation and secretion of inflammatory factors in the inflammatory environment (Fig. **6G**) (https://www.biorender.com/learn).

## 4 DISCUSSION

TFs play an important role in inflammatory response. Studies have shown that TFs that coordinate the inflammatory response mainly include *nuclear factor kB* (*NF-kB*), *AP-1*, *cAMP response element-binding protein* (*CREB*), *CCAAT/enhancer-binding protein* (*C/EBP*), and *interferon regulatory factor* (*IRF*) [22-27]. In addition, TFs involved in the progression and repair of lung injury include *NRF2*, *P53*, and *TFEB* [28-31].

In this study, we obtained the expression profiles of differentially expressed TFs in an ALI model of human lung epithelial cells through sequencing and focused on the seven most differentially expressed TFs. Intracellular verification showed that *GLI3* was significantly down-regulated in LPS-treated cells, suggesting that *GLI3* may play a role in the progression of ALI. *GLIs* are intracellular signal TFs of the Hh signaling pathway, which plays an important role in embryonic development and maintenance of the homeostatic balance of mature tissues [32-34]. This family comprises three members, *GLI1*, *GLI2* and *GLI3* [35]. *GLI3* is located on human chromosome 7p14.1. Currently, research on *GLI3* mainly focuses on brain and limb development [36, 37]. Importantly, *GLI3* plays a role in lung development in mice. The absence of *GLI3* in E13.5 mouse embryos can cause abnormalities in the size and shape of three of the five lung lobes [38]. In addition, *GLI3* is upregulated in a variety of cancers and regulates cancer cell behavior such as anchoring independent growth, angiogenesis, proliferation, and migration [37]. In acute myeloid leukemia (AML) and medulloblastoma, *GLI3* has anti-cancer effects [39, 40].

Our research showed that overexpression of GLI3 promoted cell proliferation and inhibited the increase in the levels of inflammatory factors caused by LPS, including TNF-α, IL-6, and IL-1β. In an expression profile study of LPS-treated RAW264.5 mouse macrophage, *GLI3* was found to be abnormally expressed and may be involved in the regulation of the TLR4 signaling pathway [41]. These studies suggest that *GLI3* may be involved in the regulation of congenital inflammation. Our results further confirmed the anti-inflammatory effects of GLI3 in lung cells.

Through database analysis and intracellular verification, we found that miR-143-3p induced *GLI3* mRNA degradation in lung epithelial cells, thereby inhibiting the protective effect of *GLI3* on ALI cells. MiRNAs are small, non-coding, single-stranded RNA molecules [42]. They are involved in the regulation of gene expression by reducing the stability of mRNA or inhibiting its translation [43]. As post-transcriptional regulators of gene expression, miRNAs control a variety of pathways and cell physiological processes, such as angiogenesis, cell cycle, proliferation, and apoptosis [44]. TFs can also be regulated by miRNA [45]. In recent years, many studies have shown that miRNAs are effective regulators of apoptosis and inflammatory response [46, 47]. Importantly, sepsis has been found to cause ALI/ARDS by inducing apoptosis of bronchial, alveolar epithelial cells, and vascular endothelial cells, thereby damaging the integrity of alveolar capillary membranes. Upstream regulation of GLI3 may involve a variety of signaling pathways and modification methods. Our results enrich this regulatory network.

In addition, *SFRP1* was significantly downregulated in LPS-treated cells. The Results of the double luciferase reporter assay proved that *GLI3* interacted with the promoter of *SFRP1*. Then, *SFRP1* was overexpressed in *GLI3* knockdown cells. We found that *SFRP1* overexpression promoted proliferation and inhibited the levels of TNF-α, IL-6, and IL-1β, indicating that *SFRP1,* as a transcriptional target of *GLI3,* blocked the effect of *GLI3* on LPS-treated cells. *SFRP* is a secretory glycoprotein, located in 8p12-11.1 [48]. It is encoded by *SFRP* gene and has approximately 300 amino acid residues, including a homologous N-terminal and C-terminal. *SFRP* family is an extracellular antagonist of WNT signaling pathway and consists of five members. *SFRP1* is highly expressed in lung cells [49]. However, whether *SFRP1*, as a secretory protein, is highly expressed in cells and how it participates in inflammatory response remains to be further studied. As a modulator of TGFβ1-driven fibroblast phenotypes in fibrogenesis, *SFRP1* inhibits the invasion of injury-induced myofibroblasts [50]. In another research, *SFRP1* alleviated lung fibrosis *in vivo* [51]. However, there are still unclear mechanisms of SFRP1 in lung injury that warrant further exploration.

## CONCLUSION

*GLI3* promoted the proliferation and inhibited levels of inflammatory factors by activating the transcription of *SFRP1* in ALI cell model. MiR-143-3p induced *GLI3* degradation through combining with *GLI3* mRNA. This study reveals the effect of *GLI3* and the pathogenesis of sepsis ALI, providing new potential targets for clinical diagnosis and treatment of ALI.

## Figures and Tables

**Table 1:** Primers used in this research.

**Gene**	**Forward (5'-3')**	**Reverse (5'-3')**
ID1	AAACGTGCTGCTCTACGACA	GGGGGTTCCAACTTCGGATT
HES1	ATGACAGTGAAGCACCTCCG	GAGTGCGCACCTCGGTATTA
GLI3	GGGACCAAATGGATGGAGCA	TGCAGGTGTTGTTGGACTGT
HOXB5	GGGGCAGACTCCGCAAATAT	AGATCTTGATCTGGCGCTCG
THRB	AGACGCCATCTTTGACCTGG	GTGTCACGTGGTGTTTTCGG
NFAT5	TCCCTCCTCTTCCTCCATGG	AAGACTGTGTGCCTCTTCGG
TFAP2A	AAGAGTTCACCGACCTGCTG	AGGGCCTCGGTGAGATAGTT
PRDM8	TTACACCACCTGCGACATCC	GCAGCCACATGAGACCTTCT
SETD1B	ATGTTGCTGTCTCCAGAGCC	ATGGGTGTGAGGCATCTGTG
MYEF2	TCCGGGTGGACAGCCTATTA	TCGGCCAACTCCTCCAAATC
MBTPS2	GTTGGGGTGCTCATCACTGA	TTTGGGGCTCATAGGCGATG
CCR1	GTGCCAGAAGGTGAACGAGA	CGTGAACAGGAAGAGCAGGT
PRTG	TCTTCCAGAAGCACCAGCAG	TTCACGGTGTAAGACCTGCC
SIK3	CAATTCCCCACCTTCCCTCC	GCTGTGCAAACTCTGTTGGG
KMT2A	CAACAGGGCGGAAGAAGTCT	TCTCCTTCTCCAGGGATGGG
NFAT5	TCCCTCCTCTTCCTCCATGG	AAGACTGTGTGCCTCTTCGG
|RBPMS	CAGTAGCCCTGAAGTGTGGG	GCACTATCAGGAGACGGAGC
SFRP1	GTTGGGGCCCATCAAGAAGA	TACTGGCTCTTCACCTTGCG
IRS2	CGCTGCAGCTCATGAACATC	TGGTCTCGTGGATGTTCTGC

## Data Availability

The data supporting the conclusions of this paper are included in the manuscript.

## LIST OF ABBREVIATIONS

ALI: Acute Lung Injury
ANOVA: One-Way Analysis of Variance
ARDS: Acute Respiratory Distress Syndrome
C/EBP: CCAAT/Enhancer-Binding Protein
CCK8: Cell Counting Kit
CREB: cAMP Response Element-Binding Protein
ECL: Chemiluminescence
GLI: Family Zinc Finger 3
GLI3: Glioma-Associated Oncogene
Hh: Hedgehog
IRF: Interferon Regulatory Factor
LPS: Lipopolysaccharide
NF-kB: Nuclear Factor kB
OD: Optical Density
PVDF: Polyvinylidene Fluoride
SD: Standard Deviation
SFRP1: Secreted frizzled related protein-1
TF: Transcription Factors
